# Role of Gastrointestinal Dysbiosis and Fecal Transplantation in Parkinson’s Disease

**DOI:** 10.7759/cureus.19035

**Published:** 2021-10-25

**Authors:** Rahul Jena, Ruchi Jain, Suchitra Muralidharan, Vijaya Lakshmi Yanamala, Zainab Zubair, Ketan Kantamaneni, Krishi Jalla, Mahvish Renzu, Michael Alfonso

**Affiliations:** 1 Internal Medicine, California Institute of Behavioral Neurosciences & Psychology, Fairfield, USA; 2 Diagnostic Radiology, California Institute of Behavioral Neurosciences & Psychology, Fairfield, USA; 3 Surgery, California Institute of Behavioral Neurosciences & Psychology, Fairfield, USA; 4 General Surgery, California Institute of Behavioral Neurosciences & Psychology, Fairfield, USA; 5 Surgery, Dr. Pinnamaneni Siddhartha Institute of Medical Sciences and Research Foundation, Gannavaram, IND; 6 School of Medicine, Universidad del Rosario, Bogota, COL; 7 Medicine, California Institute of Behavioral Neurosciences & Psychology, Fairfield, USA

**Keywords:** transplant, fecal microbiota, gut dysbiosis, neurodegeneration, gastrointestinal microbiome, parkinson' s disease

## Abstract

Parkinson’s disease (PD) is one of the most common neurodegenerative diseases with a high rate of morbidity. It is associated with dopaminergic neuron loss and is fairly common in the elderly population. Recently, there has been a growing interest in the role of the gut microbiome in the pathogenesis of PD and thus studies addressing the methods to modulate the microbiota are becoming increasingly popular. Fecal microbiota transplant (FMT) is one of these methods and is effective in certain intestinal and extraintestinal conditions. This review aims to talk about gastrointestinal dysbiosis and how the reconstruction of this microbiome via FMT could potentially be used as a treatment modality in the future. We went through various studies and collected data relevant to our topic from the previous five years. The studies selected include reviews, observational studies, animal studies, case reports, and some grey literature. We concluded that although it has great potential as a therapeutic modality in the future, it is limited by several factors such as variability among the results of most clinical studies and the lack of large sample sizes. Therefore, there is a need for high-quality clinical trials with larger sample sizes to gather enough clinical evidence so that FMT can qualify as a widely recommended therapeutic measure.

## Introduction and background

Parkinson’s disease (PD) is one of the most common neurodegenerative diseases of today. It contributes significantly to the current healthcare burden in terms of both mortality and morbidity. It is characterized by progressive degeneration of dopaminergic neurons in the nigrostriatal pathways [1]. At a molecular level, this occurs mainly by the formation and deposition of Lewy bodies in the central nervous system (CNS), particularly in the substantia nigra pars compacta complex. These Lewy bodies are neurotoxic and hence induce neurodegeneration, ultimately bringing about a wide array of symptoms, including bradykinesia or akinesia, muscular rigidity, tremor, gait abnormalities, dementia, depression, and sensory dysfunction [1].

It is a multifactorial disease with a very slow progression, which may vary among patients but invariably causes a wide array of complications and may even result in death. Even though PD has been studied traditionally from a neurological perspective, it is important to address that the gastrointestinal (GI) system shares a significant disease burden [1]. Patients with PD often suffer from comorbid GI dysfunction, which manifests as anything from bloating, dysphagia, and fullness to severe constipation. These symptoms vary in severity and duration when compared to a healthy population [1].

Significant research points to bidirectional communication between the gut and the brain, highlighting the role of the vagus nerve and certain other humoral mechanisms; hence, establishing an entity colloquially referred to as the gut-brain axis [2]. Reinforcing this idea, various underlying immune-mediated mechanisms have also been implicated in the pathogenesis of various neurological diseases [3]. One of the studies related to PD specifically found evidence of inflammatory cytokines like interleukin 6 (IL-6) and interleukin 1 beta (IL-1β), and cell markers such as glial fibrillary acidic protein (GFAP) and SRY-Box 10 (Sox-10) using the polymerase chain reaction, hinting towards the involvement of the gut-brain axis in the pathogenesis in PD [4]. This fact further paves the way for potential therapies to treat not only PD but a broad group of neurological conditions by exploiting this link.

It is believed that the gut microbiota plays a significant role in the pathogenesis of PD. Several strategies have been used to explore the effect of the gastrointestinal microbiota on the brain, including germ-free animals, probiotics, oral antibiotics, fecal microbiota transplant (FMT), and GI infection studies, thus pointing to the link between microbiome dysbiosis (altering gastrointestinal microbiota) and synucleinopathies such as that seen in PD [5].

In the previous decade, there has been a growing interest in the role of gut microbiota modulation as a potential source for treatment for intestinal and extraintestinal diseases. The use of the strategies mentioned earlier and the clinical implications of these interventions in treating various conditions have gained significant traction. Out of these, FMT has been one of the most promising approaches. FMT refers to the administration of a solution of fecal matter from a donor into the intestinal tract of a recipient via several different routes, including nasogastric, oral, and colonoscopy. It has already been approved as a therapeutic option in cases of recurrent *Clostridium difficile* infections, and its efficacy has been well documented [6]. A clinical trial also showed that it was an effective alternative to antibiotics even in primary *Clostridium difficile* infections. [7]. It has also shown promising results in conditions including ulcerative colitis and irritable bowel syndrome (IBS) [8,9]. Even more recently, the role of gut microbiota has been studied in neuropsychiatric disorders such as multiple sclerosis, myoclonus dystonia, chronic fatigue syndrome, depression, autism, and even idiopathic thrombocytopenic purpura, etc. [10-15]. There is good reason to believe FMT could be a potential therapeutic approach to treat PD, mainly due to the high incidence of gastrointestinal symptoms, significant gut dysbiosis, and increased gastrointestinal tract permeability [1,16]. FMT, although an excellent therapeutic measure, however, comes with its own set of problems. There is, at the moment, little solid evidence to support the benefit of FMT specifically in treating PD.

We need to keep in mind that currently available options for PD treatment are mostly limited to pharmacotherapy (mostly dopamine replacement therapies), which are associated with the resurgence of most symptoms, including tremors, dyskinesia, and psychosis. Additionally, dopamine resistance is commonly seen after long-term treatment, which is a huge problem. Such patients are poor candidates for further medical intervention. Hence, none of the currently available therapies can significantly impact the disease's progression. Thus, there is a need for novel therapies for PD. That is where FMT comes into play. In this review, we aim to assess the current literature surrounding the changes in the gastrointestinal microbiome in patients with PD and its role in the pathogenesis of PD and how modulation of this microbiota, with a special focus on FMT, affects patient outcomes.

## Review

Methods

Databases that were searched included PubMed, PubMed Central (PMC), ResearchGate, and Google Scholar. These were thoroughly searched using appropriate keywords and Medical Subject Heading (MeSH) terms to precisely point out all relevant articles related to gastrointestinal dysbiosis and the role of fecal microbiota transplants in the treatment of PD. The MeSH terms included in the search were MeSH “Parkinson disease” AND MeSH “Gastrointestinal microbiome” AND MeSH “Fecal Microbiota.” We included reviews, case reports, animal studies, and observational studies from the previous five years. We only included studies where the full-text articles could be accessed. In addition, only studies published in the English language were included. Any studies not specific to PD were excluded. Studies published in other languages and those related to other neurological disorders were also excluded.

Results

We initially found 6,780 articles on an advanced MeSH search. After applying all relevant filters, we narrowed down our results to 86 articles. All the papers that were not directly related to the research topic were manually excluded, and a total of 25 studies were included in our study.

Discussion

Several mechanisms have been proposed regarding the overall pathogenesis of PD and its association with the gut microbiome. Therefore, before addressing the various therapeutic interventions for PD, including FMT, it is crucial to delve into the association of the gut microbiome.

Gut Microbes and Parkinson’s Disease: The Link

A study by Sampson et al. from 2016 tested the role of gut bacteria in the regulation of the hallmark motor symptoms and pathophysiology of PD, more specifically alpha-synuclein (α-synuclein) dysfunction, using a mouse model [17]. They did fecal implants from PD patients into mice who ended up showing significant motor impairment, showing some value in the idea of a causal link between PD and gut microbiota [17]. They also demonstrated that the presence of specific microbes or even microbial metabolites is enough to promote α-synuclein pathology, neuroinflammatory changes, and characteristic motor and gastrointestinal dysfunction in the mouse model [17]. It has been postulated that α-synuclein deposition in PD might start in the enteric nervous system by pro-inflammatory immune activity much before the CNS symptoms manifest [18]. Hence, α-synuclein deposition in the enteric nervous system (ENS) is likely to be an etiological factor for PD. This could pave the way for immunohistochemistry-based studies to develop biomarkers to monitor disease progression [19]. Understanding the exact mechanism of α-synuclein aggregation and transport could clue us into how PD exactly spreads [20]. Factors such as increased permeability of the intestine, the vagus nerve pathway, and inflammatory cytokines that bring about significant neuroinflammation play an important role in this as well [20]. These findings can be used to highlight how α-synuclein could possibly be a biomarker for early detection of PD and predict if a person is at risk of developing it and hence initiate therapy [19,20]. A study by Santos et al. highlights vagus-mediated pathways as well as the non-vagus (bloodstream and lymphatics) pathways and emerging evidence for the same, supporting the gut to brain spread hypothesis [2].

**Figure 1:**
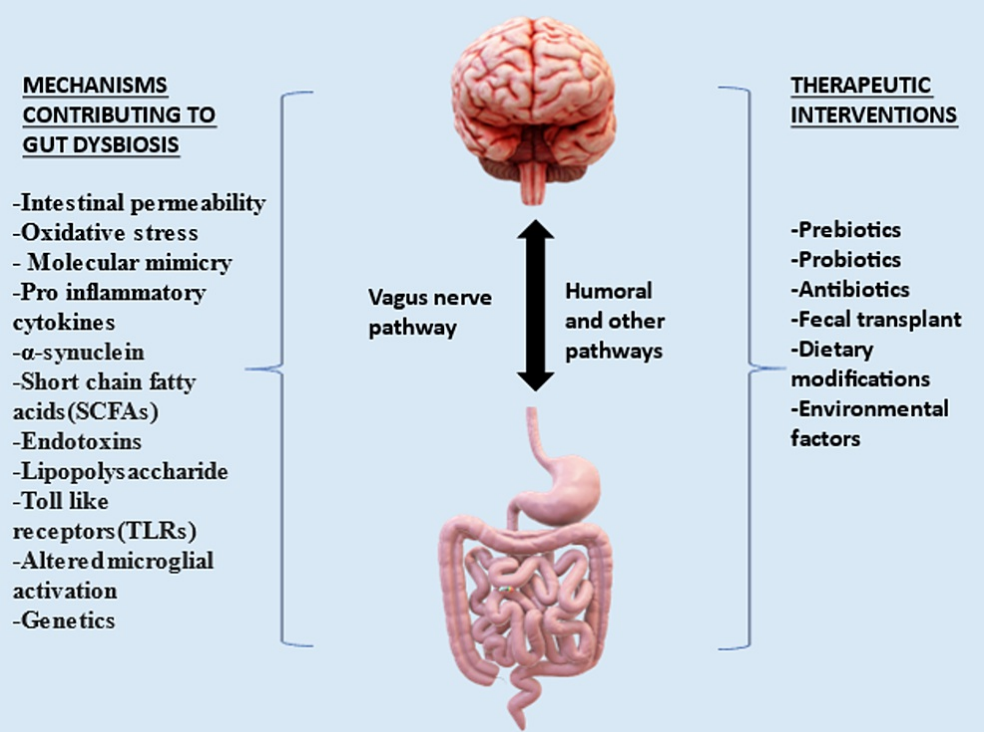
Mechanisms and therapeutic approach to intestinal dysbiosis. Original figure, made by author Rahul Jena.

The role of dysbiosis is seen in not only primary PD but also in secondary PD. Dodiya et al. conducted an interesting study to establish this [21]. It was seen that both rotenone and stress induced a significant defect in intestinal permeability and oxidative stress, leading ultimately to neuroinflammatory changes in a mouse model [21]. Other mechanisms such as molecular mimicry playing a role in the pathogenesis of PD has also gained significant traction, evidenced particularly by the growing interest in the role of bacterial amyloids [22]. However, there is a need to develop more specific causal links before going into therapeutic options. In light of all supporting evidence, altering the gut microbiome via several methods, including probiotics, prebiotics, and FMT, could definitely be a useful approach in treating PD [23]. Figure 1 shows the mechanisms and the various therapeutic approaches for GI dysbiosis.

Changes in Gastrointestinal Microbiome in Patients With PD

A detailed analysis of the gut microbiota revealed a study from 2017 by Hill-Burns et al. assessed the exact degree of gut dysbiosis in about 197 PD patients, and the data were compared to that from 130 healthy controls [24]. Statistical analysis revealed there was significant dysbiosis in patients with PD. It also showed significant alteration of various functional pathways (such as those associated with xenobiotic metabolism) [24]. Two long-term observational studies addressed the correlation of gut dysbiosis and the progression of PD in the patient [25,26]. Both assessed the exact distribution of the gut microbiome and observed a correlation between the abnormal distribution of certain gut microflora and the progression of PD symptoms [25,26].

Gorecki et al. conducted a study to analyze the gut microbiota in PD patients and mice model over-expressing α-synuclein to assess the degree of dysbiosis before the motor dysfunction set in. Significant dysbiosis was observed [27]. The major shortcomings of all these laboratory studies include mainly the presence of confounding factors and contradictory findings [28]. A study also found that the family environment also plays a significant role in gut dysbiosis, which could be explored in the future [29]. Liu et al. summarized the findings from several studies related to the exact chances in the gut microbiome compositions in patients with PD. The exact changes found in the microbiome were variable but almost all the studies basically pointed to the same thing - the presence of inflammation within the gastrointestinal tract, elevated pathogen count, and altered intestinal permeability [30].

One of the major limiting factors in these studies was a small sample size. Therefore, future studies should focus on larger numbers to gain more reliable evidence.

Role of Fecal Microbiota Transplant in Restoring Eubiosis

After considering all the evidence that points to the involvement of gut dysbiosis in the pathogenesis of PD, it is imperative to consider the various methods that bring about "eubiosis" or restore the normal flora of the gut. These methods have been studied to various degrees and include germ-free animals, infections, antibiotics, prebiotics, and FMT, as mentioned before [5,31]. Owing to the success of FMT in conditions such as recurrent *Clostridium difficile* infection (RCDI), which is also associated with gut dysbiosis, it makes sense that the coexistence of intestinal inflammation and increased intestinal permeability in patients with PD could potentially be altered with FMT, possibly ameliorating the symptoms of PD [7,16]. Hence, restoration of the native gut microbiome may potentially be a good way to approach PD.

Even though we have not established the exact relation between PD and gut microbiota dysfunction, it is worth noting that there is an appearance of gut dysfunction in the early stage of PD as well as α-synuclein deposition in both the gut and brain, supporting the gut-to-brain transmission theory [30]. This is something we could potentially exploit in the future for the treatment of the disease’s symptoms. However, there is a long way before we reach this stage in terms of the evidence currently available. Even though there is no concrete evidence linking the clinical benefit of FMT in PD, but there is some grey literature and ongoing research [32-34]. However, a few studies provide corroborative experimental evidence for some value of FMT in patients with PD [35,36]. A case report by Huang et al. of a 71-year-old PD patient with intractable constipation showed that the time spent in defecation after FMT was reduced from over 30 minutes to under five minutes. This was maintained for three months until the end of the follow-up period. There was also general improvement of the motor symptoms such as tremors, which disappeared within one week, although there was some recurrence with reduced severity after two weeks [35]. The other study assessed the impact of FMT on 11 patients with PD and constipation. All these patients were reported to have complete remission of constipation and very mild side effects, none of which hindered their participation in the study. Taxonomic analysis revealed a decrease in the community abundance of fecal microbiota, and the microbial diversity was lower in patients before they were treated with FMT [36]. Xue et al. also conducted a study involving 15 patients with PD, who received FMT via two different routes (10 by colonoscopy and five by a nasogastric tube). Interestingly, this study showed the clear superiority of colonoscopy over the nasogastric method, particularly for non-motor symptoms such as anxiety, depression, and sleep quality. Furthermore, other than some mild adverse effects (in five patients), no significant adverse effect was noted in the follow-up and extended follow-up period. It was concluded that colonoscopic FMT is probably more efficacious, particularly due to its direct effect on the colonic microbiota [37].

Although we have moved closer to the identification of specific strains and underlying mechanisms implicated in PD-associated gut dysbiosis, there remain too many unanswered questions and still too many variables to address, such as age, sex, ethnicity, use of medications, diet, and immune status, that have not been taken into account in most studies [38]. Moreover, studies mostly point to obvious clinical benefits through FMT, majorly in cases of PD associated with severe constipation only. More detailed studies with due consideration given to other variables and also confounders are required at the moment. There is also a need to explore the exact mechanism by which FMT restores eubiosis and the exact changes in the gastrointestinal microbiota before and after FMT [38,39]. Only then can we move toward developing a standardized protocol for FMT. As mentioned earlier, this can only be achieved with extensive standardized clinical trials with large sample sizes. Only then can sufficient data be acquired to move FMT forward in therapeutic implementation. All the review articles that were included in our study are summarized in Table 1. All the animal studies, observational studies, and a case report that were included in our study are summarized in Table 2.

**Table 1:** Summary of selected review articles. FMT: fecal microbiota transplant; PD: Parkinson's disease; GIT: gastrointestinal tract; CNS: central nervous system.

Author	Study type	Objective of study	Relevant conclusion
Parashar et al. [31]	Traditional review	To assess the association between gut dysbiosis and PD, as well as the therapeutic options available.	Gut dysbiosis is most likely implicated in PD leading to the motor fluctuations of PD. Restoring eubiosis may be a viable option.
Perez-Pardo et al. [18]	Traditional review	To review literature associating gut dysbiosis acting as an inflammatory trigger for PD.	The pathogenesis of PD probably begins in the gut and later spreads to the CNS, which may manifest in the form of motor symptoms.
Scheperjans et al. [20]	Traditional review	To address current opinions on the gut-brain axis with a special focus on α-synuclein pathology in PD and potential biomarkers.	α-synuclein deposition in both GIT and CNS is an important feature of PD. Immunohistochemistry may have the potential to detect and use it as a biomarker for the progression of PD in the future.
Fitzgerald et al. [19]	Traditional review	To explore the current literature surrounding the role of the microbiota in the pathogenesis of PD with a special focus on the role of α-synuclein.	PD may possibly originate from the gut and detection of α-synuclein here could potentially be used as a biomarker to track disease progression.
Gorecki et al. [27]	Traditional review	To assess the degree of dysbiosis in mice model over-expressing α synuclein.	Significant dysbiosis in the form of an abundance of Gammaproteobacteria coupled with a decrease of Clostridia and Bacteroides spp.
Haikal et al. [28]	Traditional review	To assess the findings from the various studies and reflect on the future of microbiome studies in PD research.	Review the findings of different studies and assess the role of gut microbiome studies in PD research.
Santos et al. [2]	Traditional review	To assess the association between PD and the gut-brain axis, highlighting evidence of gut dysbiosis and its role in the pathogenesis of PD.	Dysbiosis plays a very important role in PD potentially. Preventive measures to ensure a healthy gut microbiome can go along the way to ensure less risk of developing PD.
Miraglia et al. [22]	Traditional review	To address the link between dysbiosis and PD with focus given to molecular mechanisms such as molecular mimicry.	The gut microbiome may significantly contribute to the neurotoxicity of PD even on a molecular level.
Dutta et al. [5]	Traditional review	To evaluate the role of probiotics and FMT in PD.	FMT and probiotics could potentially be used to treat FMT.
Lorente-Picón et al. [38]	Traditional review	To assess the status of different strategies used for the treatment of PD including dietary interventions and FMT.	Microbiota-based therapeutic strategies including FMT can be potentially useful in PD patients.
Liu et al. [30]	Traditional review	To evaluate the role of gut microbiota in the etiology of PD and assess the possible therapeutic options from this association.	Antibiotics, probiotics, prebiotics, or FMT could normalize the gut ecosystem and possibly improve brain functions in PD patients.
Huang et al. [23]	Traditional review	To explore the role of intestinal dysbiosis in PD and highlight various mechanisms including the microbes, their metabolites, inflammation, damage to the gut barrier, and α-synuclein pathology.	There is a significant gut dysbiosis in a patient with PD.
Kang et al. [39]	Traditional review	To evaluate the latest available research on PD and its association with the gut microbiome, and to explore FMT as a future potential therapy.	FMT is a promising therapeutic measure for the treatment of PD in the future.

**Table 2:** Summary of observational studies, animal studies, and a case report included in our study. FMT: fecal microbiota transplant; PD: Parkinson's disease.

References	Study type	Study characteristics	Objective of study	Relevant conclusion
Sampson et al. [17]	Animal study	Mouse models, overexpressing α-synuclein were used to study the motor impairment and pathophysiology of PD, by comparing them to controls.	To implicate gut bacteria in the regulation of the motor symptoms and pathophysiology of PD using a mouse model as well as associated α-synuclein dysfunction.	The presence of microbes or their metabolites can induce α-synuclein dysfunction neuroinflammatory changes, and characteristic motor and gastrointestinal dysfunction in the mouse model.
Hill-Burns et al. [24]	Observational study	Gut dysbiosis in 197 PD patients and 130 healthy controls were analyzed.	To determine if PD involves dysbiosis of the gut microbiome, and identify the microbial taxa and functional pathways affected.	An altered abundance of *Bifidobacteriaceae, Christensenellaceae,* *Lachnospiraceae,* *Lactobacillaceae,* *Pasteurellaceae, and* *Verrucomicrobiaceae* families was observed. Significant alteration in various functional pathways.
Minato et al. [26]	Observational study	36 PD patients	To assess whether gut dysbiosis is clinically correlated with the progression of PD.	Significant dysbiosis was observed.
Cilia et al. [25]	Observational study	39 de novo PD patients	To investigate whether gut microbiota in early untreated PD may predict motor and non-motor symptoms over a three-year period.	Significant microbial abnormalities with particularly reduced abundance of Roseburia and Ruminococcaceae and Actinobacteria at baseline lead to worse neurological functional outcomes in the three-year follow-up period.
Dodiya et al. [21]	Animal study	Gut dysfunction was studied in mice subjected to chronic stress and low dose rotenone, and compared to healthy controls	Intestinal hyperpermeability and gut dysbiosis combined can exacerbate stress and rotenone-induced PD in a mouse model.	Pro-inflammatory intestinal milieu, oxidative stress, and α-synuclein were found in these mice pointing to the link between dysfunction of the gut microbiome and PD symptoms.
Xue et al. 2020 (37)	Preliminary study	15 patients with PD, who received FMT (10 by colonoscopy and five by a nasogastric tube)	To assess the efficacy and safety of FMT on PD patients via two separate routes.	Colonoscopic FMT showed better results than nasogastric FMT. Both motor and non-motor symptoms were well controlled in the former. Mild adverse effects were seen overall.
Zhang et al. [29]	Observational study	63 PD patients, 63 healthy spouses (HS), and 74 healthy people (HP)	To assess the association between gut microbiota and disease progression in PD patients and the effect of the family environment.	Altered gut microbiota is observed in PD patients and these are also influenced by the family environment.
Kuai et al. [36]	Prospective, single study	11 PD patients with constipation	To assess the safety of FMT to treat PD-associated gastrointestinal dysfunction.	Remission of constipation in all patients, which might be related to increased microbial abundance. FMT is hence a good choice for PD treatment with gastrointestinal dysfunction.
Huang et al. [35]	Case report	A 71-year-old male patient with PD from seven years (with marked constipation for more than three years)	To assess the therapeutic role of FMT in the case of PD with refractory constipation.	Improvement in both constipation (gastrointestinal dysfunction) and tremors (motor symptoms). Hence, FMT (as a form of gut microbiota reconstruction) shows therapeutic benefits.

Limitations

This review is limited to studies in the English language, so we may have missed valuable studies published in other languages. In addition, the studies before 2016 are excluded, which may also have caused a similar limitation. The most significant limiting factor of most studies included in this review is the small sample size, which potentially limits the generalizability of the results published.

## Conclusions

PD is a multifactorial disease and is closely associated with changes in the GI microbiome. In this review, we explored the various mechanisms that contribute to GI dysbiosis and concluded that dysbiosis is very likely to be involved in the pathogenesis of PD through mechanisms that include intestinal barrier disruption, inflammation, oxidative stress, decreased dopamine production, and molecular mimicry. Assessing the exact changes in the GI microbiome and their modulation is also an important consideration. We also assessed various treatment strategies with a focus on the role of FMT. Although it has shown successful outcomes, it has certain weaknesses in terms of being a widely accepted therapeutic approach. The main limiting factor of gathered knowledge was a small sample size and variability in findings from most laboratory studies. We conclude that FMT seems to be a good treatment strategy, especially since it is a simple procedure and is fairly cost-effective. However, future studies should aim to focus on safety, routes of administration, standardized protocols, and the adverse effect profile of FMT as well.
